# Mapping the Key Residues within the Porcine Reproductive and Respiratory Syndrome Virus nsp1α Replicase Protein Required for Degradation of Swine Leukocyte Antigen Class I Molecules

**DOI:** 10.3390/v14040690

**Published:** 2022-03-26

**Authors:** Yuanyuan Liu, Peng Gao, Lei Zhou, Xinna Ge, Yongning Zhang, Xin Guo, Jun Han, Hanchun Yang

**Affiliations:** Key Laboratory of Animal Epidemiology of the Ministry of Agriculture and Rural Affairs, College of Veterinary Medicine, China Agricultural University, Beijing 100193, China; liuyuanyuan007@126.com (Y.L.); penggao@cau.edu.cn (P.G.); leosj@cau.edu.cn (L.Z.); gexn@cau.edu.cn (X.G.); zhangyongning@cau.edu.cn (Y.Z.); guoxin@cau.edu.cn (X.G.)

**Keywords:** porcine reproductive and respiratory syndrome virus (PRRSV), nonstructural protein 1α (nsp1α), swine leukocyte antigen class I (SLA-I), degradation, reverse genetics, immune escape

## Abstract

The nonstructural protein 1α (nsp1α) of the porcine reproductive and respiratory syndrome virus (PRRSV) has been shown to target swine leukocyte antigen class I (SLA-I) for degradation, but the molecular details remain unclear. In this report, we further mapped the critical residues within nsp1α by site-directed mutagenesis. We identified a cluster of residues (i.e., Phe17, Ile81, Phe82, Arg86, Thr88, Gly90, Asn91, Phe94, Arg97, Thr160, and Asn161) necessary for this function. Interestingly, they are all located in a structurally relatively concentrated region. Further analysis by reverse genetics led to the generation of two viable viral mutants, namely, nsp1α-G90A and nsp1α-T160A. Compared to WT, nsp1α-G90A failed to co-localize with either chain of SLA-I within infected cells, whereas nsp1α-T160A exhibited a partial co-localization relationship. Consequently, the mutant nsp1α-G90A exhibited an impaired ability to downregulate SLA-I in infected macrophages as demonstrated by Western blot, indirect immunofluorescence, and flow cytometry analysis. Consistently, the ubiquitination level of SLA-I was significantly reduced in the conditions of both infection and transfection. Together, our results provide further insights into the mechanism underlying PRRSV subversion of host immunity and have important implications in vaccine development.

## 1. Introduction

Porcine reproductive and respiratory syndrome virus (PRRSV) is an enveloped, positive-stranded RNA virus in the genus *Porartevirus* of the family *Arteriviridae* in the order *Nidovirales* [1,2]. This agent mainly causes reproductive failure in sows and severe respiratory distress in piglets with sometimes high morbidity and mortality [3,4]. Ever since its first emergence in the late 1980s in both North America and Europe, PRRSV has remained a major threat to the worldwide swine industry [5,6,7]. The currently available PRRSV modified live-attenuated vaccines (MLVs) are generally effective against the challenge of homologous viruses but fail to induce sterilizing immunity or to provide efficient cross-protection against heterologous strains [3,8,9,10]. The failure of viral clearance from hosts is largely attributed to the intrinsic properties of PRRSV.

Evasion or subversion of host immunity is a prominent feature of PRRSV [11,12,13]. This property often leads to dysregulation of innate immunity [14,15], delayed and low-level induction of neutralizing antibodies [16,17], and inadequate and poor quality of cytotoxic T lymphocyte (CTL) responses [18,19]. Clinically, PRRSV infection is characterized by persistent infections in swine herds, which allows for further selection of escape mutants due to the accumulative mutations or recombination [7]. Clearly, a better understanding of the viral immune evasion mechanisms is critically needed for development of better vaccines against PRRSV.

The CTL responses are a critical line of host defenses in containing intracellular pathogens [20]. Antigen peptide presentation mediated by the major histocompatibility complex class I (MHC-I) molecule is a key step for CD8^+^ T-cell activation [21,22]. Thus, the viruses, especially those capable of establishing persistent or chronic infections, such as the human immunodeficiency virus (HIV) and the mouse norovirus (MNV), have evolved intricate means to manipulate the MHC-I presentation pathway, thus limiting MHC-I-mediated cellular immunity [21,23]. In pigs, MHC-I is termed as swine leukocyte antigen class I (SLA-I), and it is composed of a heavy chain (HC) and a light chain (β2m-miroglobulin (β2m)) [24]. It is well documented that PRRSV infection reduces the accumulation of SLA-I on the cell surface of porcine macrophages and dendritic cells [25,26]. This is attributed to several viral factors. Du et al. were the first to report that PRRSV nonstructural protein 1α (nsp1α) is capable of targeting SLA-I for degradation via the ubiquitin–proteasomal pathway [27]. Subsequently, PRRSV nsp2TF was found to be associated with SLA-I downregulation [28] and then was the replicase protein nsp4 that was linked to the downregulation of β2m at the mRNA level by binding to the B2M promoter to suppress the transcription [29]. Despite these efforts, more molecular details await to be discovered concerning the viral modulation of CTL responses.

**Figure 1:**
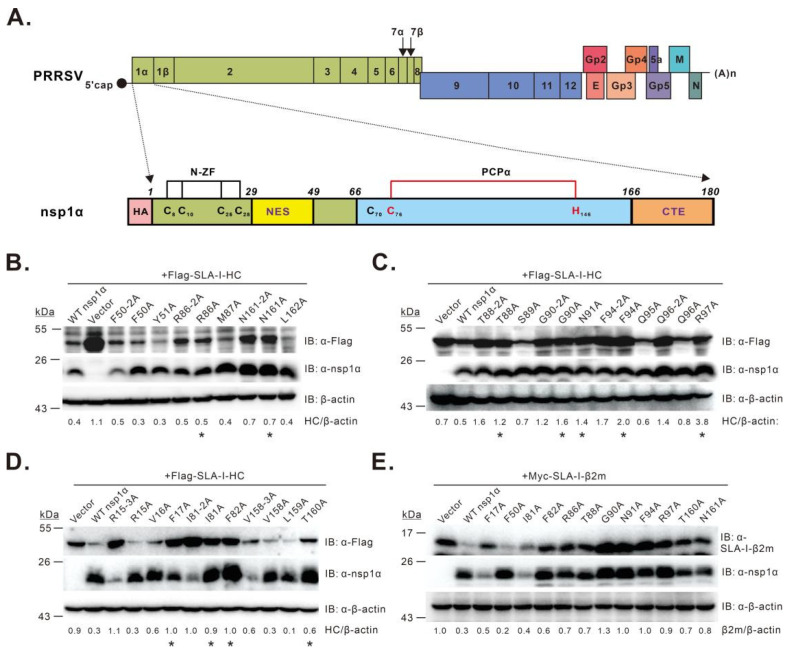
Identification of residues critical for PRRSV nsp1α-mediated degradation of SLA-I. (**A**) structure organization of the PRRSV genome and nsp1α; (**B**–**E**) screening of nsp1α residues necessary for SLA-I degradation by co-transfection assay. HEK 293T cells were transfected to express FLAG-SLA-I-HC (**B**–**D**) or Myc-β2m (**E**) in combination with HA-nsp1α or its mutants. At 36 h post-transfection, the cells were subject to Western blot analysis with antibodies to FLAG, SLA-I-β2m, nsp1α, or β-actin. Asterisk (*) indicates the mutants that were selected for further analysis. The data are representative of results from three independent experiments. The relative abundance of SLA-I was normalized against β-actin, and the ratio is shown at the bottom of the blots.

In this study, we investigated the molecular mechanism of PRRSV nsp1α-mediated SLA-I degradation but with a specific focus on nsp1α itself. This viral replicase protein has a size of 180 amino acids and contains three discernible domains: an N-terminal zinc finger domain (N-ZF domain; Met1 to Glu65) composed of a conserved signature motif Cys8–Cys10–Cys25–Cys28; a papain-like cysteine protease domain (PCPα domain; Pro66 to Gln166) using residues Cys76 and His146 as the catalytic dyad; a C-terminal extension region (CTE; Arg167 to Met180) (Figure 1A) [30,31]. We previously showed that an intact structure of nsp1α, but not the protease activity, is necessary for SLA-I degradation [27]. In this report, we went further to dissect the critical residues of nsp1α in both transfection and infection conditions. Our results revealed the residue Gly90 is a promising target for vaccine development.

## 2. Materials and Methods

### 2.1. Cells, Virus, and Infection

Porcine pulmonary alveolar macrophages (PAMs) were prepared as previously described [32] and maintained at 37 °C with 5% CO_2_ in RPMI 1640 medium (Thermo Fisher Scientific, Waltham, MA, USA) supplemented with 10% (*v*/*v*) fetal bovine serum (FBS) (Thermo Fisher Scientific, Waltham, MA, USA), 50 U/mL penicillin, and 50 mg/mL streptomycin. MARC-145, Vero, and HEK 293T cells were all cultured in Dulbecco’s modified Eagle’s medium (DMEM) (Thermo Fisher Scientific, Waltham, MA, USA) supplemented with 10% FBS and penicillin (50 U/mL) and streptomycin (50 mg/mL) in a humidified incubator with 5% CO_2_ at 37 °C. The HP-PRRSV strain, JXwn06 (GenBank accession no: EF641008), used in this study has been described previously [33]. In infection condition, PAMs or MARC-145 cells were grown at 37 °C with 5% CO_2_ in RPMI 1640 medium or DMEM supplemented with 2% FBS and penicillin (50 U/mL) and streptomycin (50 mg/mL).

### 2.2. Enzymes, Antibodies, and Chemicals

Restriction enzymes were all purchased from New England Biolabs Inc. (Ipswich, MA, USA). Mouse anti-actin (#A5441) monoclonal antibody (mAb) was from Merck KGaA (Darmstadt, Germany). Mouse anti-FLAG (#M185) mAb was from Medical & Biological Laboratories (MBL, Nagoya, Japan). Rabbit anti-HA (#3724) mAb was from Cell Signaling Technology (CST, Boston, MA, USA). Mouse anti-ubiquitin mAb (#BE4002) was from Bioeasy (Beijing, China). Mouse anti-N protein mAb was kindly provided by Ping Jiang (Nanjing Agriculture University, Nanjing, China). Rabbit anti-nsp1α protein polyclonal antibody (pAb) was kindly provided by Changjiang Weng (Harbin Veterinary Research Institute, Chinese Academy of Agricultural Sciences, Haerbin, China). Horseradish peroxidase (HRP)-conjugated goat anti-mouse pAb (#ZB-2305) and HRP-conjugated goat anti-rabbit pAb (#ZB-2301) were obtained from ZSGB-BIO (Beijing, China). Both Alexa Fluor 488-conjugated goat anti-mouse IgG(H+L) F(ab′)2 fragment (#A-11070) and Alexa Fluor 568-conjugated goat anti-rabbit IgG(H+L) F(ab′)2 fragment (#A-11019) were purchased from Thermo Fisher Scientific Inc (Waltham, MA, USA). The mouse anti-SLA-I mAb JM1E3 (#MCA2261GA) and mouse IgG1 antibody (#MCA928) used for flow cytometry were from AbD Serotec (Kidlington, UK). Rabbit anti-SLA-I-HC pAb, rabbit anti-β2m pAb, mouse anti-SLA-I-HC mAb, and mouse anti-β2m mAb were prepared in our laboratory [34]. MG132 (#S2619) was purchased from Selleckchem (Houston, TX, USA).

### 2.3. Plasmid Construction

Plasmids pHA-nsp1α, pFLAG-SLA-I-HC, and pMyc-SLA-I-β2m have been described previously [27]. The plasmid pHA-nsp1α served as the template for the construction of a series of nsp1α mutants using a fast mutagenesis system (TransGen, Beijing, China) to introduce amino acid substitutions. All recombinant plasmids were constructed by standard molecule biology techniques and confirmed by DNA sequencing.

### 2.4. Site-Directed Mutagenesis of PRRSV JXwn06 nsp1α and Virus Rescue

The plasmid pCMV-JXwn06, containing the full-length cDNA clone of PRRSV strain JXwn06, has been described previously [35]. To perform mutagenesis, the fragment A (bases 1–4818) containing the nsp1α-coding region was PCR-amplified from pCMV-JXwn06, using the upstream primer (5′-CAGAGCTGGTTTAGTATTTAAATACCGTCATGACGTATAGGTGT-3′) containing the *Swa* I recognition sequence (underlined) and the downstream primer (5′-CCTCCCCCTGAAGGCTTCGAAATTTGCCTGATCTTTAGTCCATT-3′) containing the *Xho* I recognition sequence (underlined), and then cloned into the plasmid Pjet1.2/blunt (Thermo Fisher Scientific, Waltham, MA, USA) to construct a shuttle plasmid Pjet1.2-A. Mutagenesis of specific nsp1α nucleotides was then carried out using a fast mutagenesis system (TransGen, Beijing, China). After confirmation by DNA sequencing, fragment A was cut off and inserted back into the PRRSV infectious clone backbone.

For virus recovery, MARC-145 cells seeded on 6-well plates at a confluency of 70–80% were transfected with the infectious clone plasmids by Lipofectamine LTX (Thermo Fisher Scientific, Waltham, MA, USA) according to the manufacturer’s protocol. The virus-induced cytopathic effect (CPE) was monitored daily. The rescued viruses were passaged 3 times in MARC-145 cells and then examined by indirect immunofluorescence assay (IFA) using the anti-N mAb SDOW17 (Rural Technologies, Brookings, SD, USA). The mutated sites were confirmed by sequencing the genome of the third-passage viruses as described previously [33].

### 2.5. Growth Properties of Viral Mutants

MARC-145 cells and PAMs were infected with the indicated viruses at the multiplicity of infection (MOI) of 0.1. After incubation for 1 h at 37 °C with 5% CO_2_, MARC-145 cells and PAMs were treated as described previously [35]. The supernatants or the cells were collected at indicated times, and the virus titers were determined using endpoint dilution assays as previously described [36].

### 2.6. Confocal Microscopy

Vero cells or PAMs seeded on coverslips in 12-well plates were transfected with indicated plasmid or infected with the indicated virus at an MOI of 1.0. At 24 h post-transfection or at 12 h post-infection, the cells were fixed with 3.7% paraformaldehyde for 10 min at room temperature (RT), washed with 1X phosphate-buffered saline (PBS) 3 times, permeabilized with 0.1% Triton X-100/2% bovine serum albumin (BSA) for 10 min, and blocked with 2% BSA/PBS for 30 min (RT). The cells were then incubated with proper primary antibodies for 1 h in a humid chamber (RT) and washed with 1X PBS 3 times. Afterwards, the cells were incubated with appropriate secondary antibodies, including Alexa Fluor 568-conjugated goat anti-rabbit IgG(H+L) F(ab′)2 fragment and Alexa Fluor 488-conjugated goat anti-mouse IgG(H+L) F(ab′)2 fragment, for another 1 h (RT). Nuclear DNA was stained with 4′,6-diamidino-2-phenylindole (DAPI) (Thermo Fisher Scientific, Waltham, MA, USA). The images were captured using a Nikon A1 confocal microscope and processed using Image J.

### 2.7. Flow Cytometry Analysis

The method for flow cytometry analysis to examine the cell surface expression of SLA-I molecules has been described previously [27]. Briefly, PAMs were seeded into six-well plates at a density of 6 × 10^5^ cells/well and gently washed with RPMI 1640 medium to remove the unattached cells. The cells were then mock infected with RPMI 1640 or infected with the indicated virus at an MOI of 1.0. At 12 h post-infection, the cells were dissociated from the plates with 0.1% EDTA and washed twice immediately with ice-cold 1X PBS containing 1% BSA. The cells were then incubated with mouse anti-SLA-I mAb JM1E3 (2 μg/mL) in 1X PBS containing 1% BSA at 4 °C for 30 min, followed by incubation with Alexa Fluor 488-conjugated goat anti-mouse IgG(H+L) F(ab′)2 fragment (1:1000) for 30 min at 4 °C. Meanwhile, a mouse IgG antibody was used as the isotype control. A total of 2 × 10^4^ cells were analyzed by fluorescence-activated cell sorter (FACS) analysis, and the cell surface expression level of the SLA-I molecules was presented as the mean fluorescence intensity (MFI).

### 2.8. Immunoprecipitation and Ubiquitination Assays

For transfection-based assays, HEK 293T cells seeded in six-well plates were transfected to express SLA-I-HC or β2m and ubiquitin with or without wild-type (WT) nsp1α or nsp1α-G90A. At 18–24 h post-transfection, the cells were treated with 10 μM MG132 for 4 h. In assays using infected cells, PAMs seeded in six-well plates were mock-infected with RPMI 1640 or infected with indicated viruses at an MOI of 1.0. At 4–6 h post infection, MG132 was added at a final concentration of 5 μM and maintained for 8 h. Harvested cells were washed 3 times with ice-cold 1X PBS and then lysed in ice-cold lysis buffer (50 mM Tris-HCl (pH7.4), 1 mM EDTA, 150 mM NaCl, 5 mM MgCl_2_, 10% glycerol, and 1% Triton X-100) supplemented with 1X cocktail (Merck) for 30 min with gentle rotation. Following centrifugation at 12,000 rpm for 30 min at 4 °C, the supernatants were transferred to a fresh tube, precleared with protein A/G magnetic beads (Thermo Fisher Scientific, Waltham, MA, USA, #88802) for 2 h at 4 °C, and then incubated with indicated antibodies for 12 h at 4 °C. The SLA-I complexes were captured with protein A/G magnetic beads for 2 h at RT. The beads were washed with 1X Tris-buffered saline (TBS) containing 0.05% Tween-20 detergent 4 times and purified water once. The immunoprecipitants were separated from the beads by low-pH elution buffer (0.1 M glycine, pH 2.0), neutralized with neutralization buffer (1.0 M Tris-HCl, pH 8.0) and subject to Western blot analysis with proper antibodies.

### 2.9. Western Blot Analysis

The extracted total proteins were quantified by a bicinchoninic acid (BCA) protein assay kit (Thermo Fisher Scientific, Waltham, MA, USA). The protein samples were resolved by SDS-PAGE with 12% polyacrylamide gel, transferred onto a 0.2 μm PVDF membrane, blocked with PBST (PBS with 0.05% Tween-20 detergent) containing 5% skim milk powder for 1 h at RT, and then probed with thee appropriate primary antibodies for 2 h at RT. The membranes were washed 3 times with PBST, incubated with the appropriate HRP-conjugated secondary antibodies at a dilution of 1–10,000 for 1 h at RT, washed again 3 times with PBST, and then developed using the ECL Western blot system (Thermo Fisher Scientific, Waltham, MA, USA).

### 2.10. Statistical Analysis

Statistical analyses were performed using the two-way analysis of variance (ANOVA) test in GraphPad Prism version 5.0 software (San Diego, CA, USA). Differences were considered statistically significant at a *p*-value < 0.05.

## 3. Results

### 3.1. Screening of nsp1α Residues Critical for Inducing SLA-I Degradation

To identify critical residue(s) for nsp1α function, we took the alanine scanning approach by site-directed mutagenesis. We excluded the residues that are important for maintaining the structure of individual domains, such as the six core residues in the N-ZF, PCPα, and CTE domains mentioned above; those (i.e., Cys70, Cys76, His146, and Met180) that tetrahedrally coordinate with C-terminal zinc ions in the nsp1α 3D structure [30]; those (i.e., Glu69 and Asn143) that can form an elaborate hydrogen bond to stabilize the Cys76–His146 dyad [30]. The mutagenesis blocks ranged from 2 to 6 amino acids (aa) in length. As a result, we constructed a total of 33 nsp1α mutants and tested the mutational effects with a co-transfection assay. The HEK 293T cells were transfected to co-express the mutants together with FLAG-SLA-I-HC, followed by Western blot analysis. The initial screening (Table S1) identified 12 mutants, including P12-6A, V18-6A, Q40-5A, F50-5A, L78-5A, P83-5A, T88-5A, N93-5A, G109-5A, V138-4A, T156-5A, and N161-5A, that exhibited reduced activity to degrade SLA-I-HC compared to WT nsp1α. Next, we further shortened the block size to 2–3 amino acids, resulting in 27 additional nsp1α mutants. Ten mutants (i.e., R15-3A, F50-2A, I81-2A, R86-2A, T88-2A, G90-2A, F94-2A, Q96-2A, V158-3A, and N161-2A) displaying impaired activity were therefore selected for the third-round analysis (Table S2). Accordingly, a total of 20 mutants carrying single substitutions were engineered. The results revealed 11 mutants (i.e., F17A, I81A, F82A, R86A, T88A, G90A, N91A, F94A, R97A, T160A, and N161A) with decreased activity (Figure 1B–D). The mutational effect of these mutations was also tested on SLA-I-β2m. The results revealed that these mutants showed variable extent of degradation activity on the substrate SLA-I-β2m (Figure 1E). As a positive control, nsp1α-F50A retained the ability to induce SLA-I-β2m degradation (Figure 1E). Overall, we have identified a cluster of residues that are critical for nsp1α to modulate SLA-I abundance.

### 3.2. Characterization of the nsp1α Mutants with Decreased Degradation Activity

**Figure 2:**
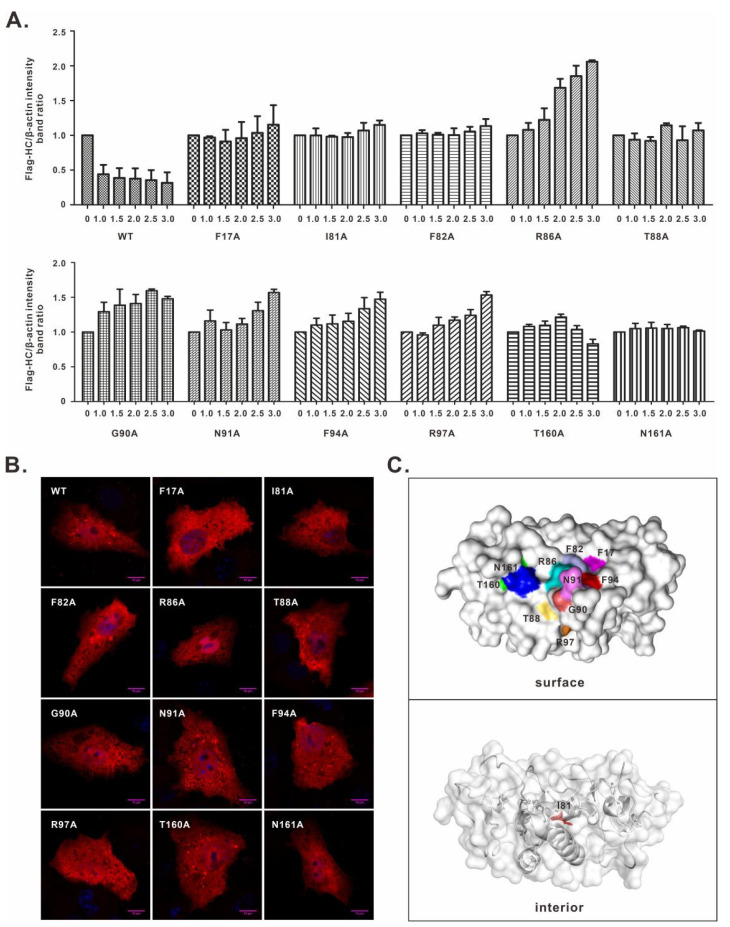
Characterization of the key residues involved in nsp1α-induced SLA-I degradation. (**A**) Dose-dependent effect of nsp1α or its derivatives on inducing degradation of FLAG-SLA-I-HC. HEK 293T cells were transfected to co-express FLAG-SLA-I-HC in combination with an increasing amount (i.e., 0, 1.0, 1.5, 2.0, 2.5, and 3.0 μg) of HA-nsp1α or its derivatives. At 36 h post-transfection, the cells were subject to Western blot analysis. The result showed the band intensity ratio between FLAG-SLA-I-HC and β-actin. (**B**) Vero cells were transfected to express WT HA-nsp1α or its mutants. At 24 h post-transfection, the cells were fixed, permeabilized, and stained with anti-HA antibody. DAPI was used to stain cell nuclei. The images were acquired by a Nikon A1 confocal microscope and are representative of three independent experiments. (**C**) Structural distribution of the nsp1α critical residues. The 3D structure of nsp1α is shown in surface and cartoon modes, and the residues are highlighted in different colors.

We next tested the mutational effect in a dose-dependent manner. HEK 293T cells were transfected to co-express FLAG-SLA-I-HC (1.0 μg) with WT nsp1α or its derivatives at different doses (0, 1.0, 1.5, 2.0, 2.5, and 3.0 μg). Western blot analysis showed that WT nsp1α induced SLA-I-HC degradation in a dose-dependent manner. In contrast, all 11 nsp1α mutants lost the ability to do that even with increased doses (Figure 2A and Figure S1). We also looked into the cellular localization of these mutants. In transfected cells (Figure 2B), all the mutants exhibited a similar diffusive distribution pattern as to WT nsp1α (Figure 2B), suggesting that the decreased activity was less likely due to the alteration of localization. Structurally, all the residues, except I81, were on the surface of the nsp1α molecule, and they were located in a relatively concentrated region (Figure 2C), indicating they were maybe involved in some kind of interaction.

### 3.3. Recovery and Growth Kinetics of nsp1α Mutant Viruses

**Figure 3:**
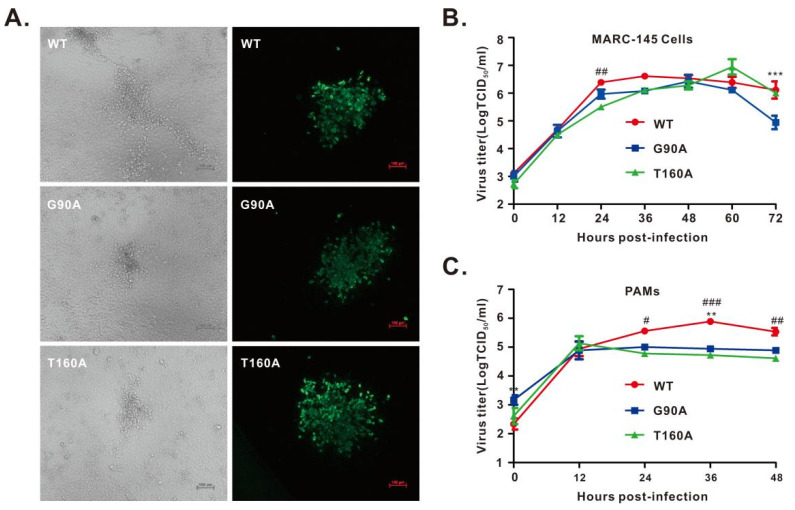
Growth kinetics of the rescued viruses. (**A**) MARC-145 cells were infected with indicated viruses at an MOI of 0.1 for 48 h. The left panel shows virus-induced CPE, and the right panel shows the IFA staining of virus-infected cells with an antibody to PRRSV N protein. The representative images were taken by an inverted Nikon microscope. (**B**,**C**) Growth kinetics of the rescued viruses in MARC-145 cells (**B**) and PAMs (**C**) at an MOI of 0.1. The total viruses were titrated in MARC-145 cells by an endpoint dilution assay. Asterisks indicate a significant difference in the virus titers between WT and G90A (** *p* < 0.01; *** *p* < 0.001). Pound symbols (#) represent a significant difference in the virus titers between WT and T160A (# *p* < 0.05; ## *p* < 0.01; ### *p* < 0.001). The data are shown as the mean ± SD of three independent experiments.

To investigate the mutational effect in the context of PRRSV infection, single-point mutations of the above residues were introduced into the DNA-launched infectious cDNA clone of PRRSV strain JXwn06 [35]. After confirmation by DNA sequencing, both WT and mutant infectious cDNA clones were transfected into MARC-145 cells. For each mutant, we chose 2–3 independent clones for virus recovery. Only two mutant viruses (i.e., G90A and T160A) were successfully recovered, as evidenced by CPE and IFA with antibodies to N protein (Figure 3A; Table S3). In contrast, the other nine mutants (i.e., F17A, I81A, F82A, R86A, T88A, N91A, F94A, R97A, and N16A) were lethal to the virus. We could not recover the viruses even after 3–4 rounds of blind passages in MARC-145 cells; detection by real-time PCR also gave negative results (data not shown). The two viable mutants of passage 3 (P3) were chosen for growth kinetics analysis in both MARC-145 cells and primary PAMs. They showed relatively similar growth properties to the parental virus JXwn06 in MARC-145 cells but exhibited a reduced growth rate in PAMs by approximately half a log at 12 h post-infection (Figure 3C).

### 3.4. Co-Localization Analysis of nsp1α Mutants with SLA-I in PRRSV-Infected PAMs

**Figure 4:**
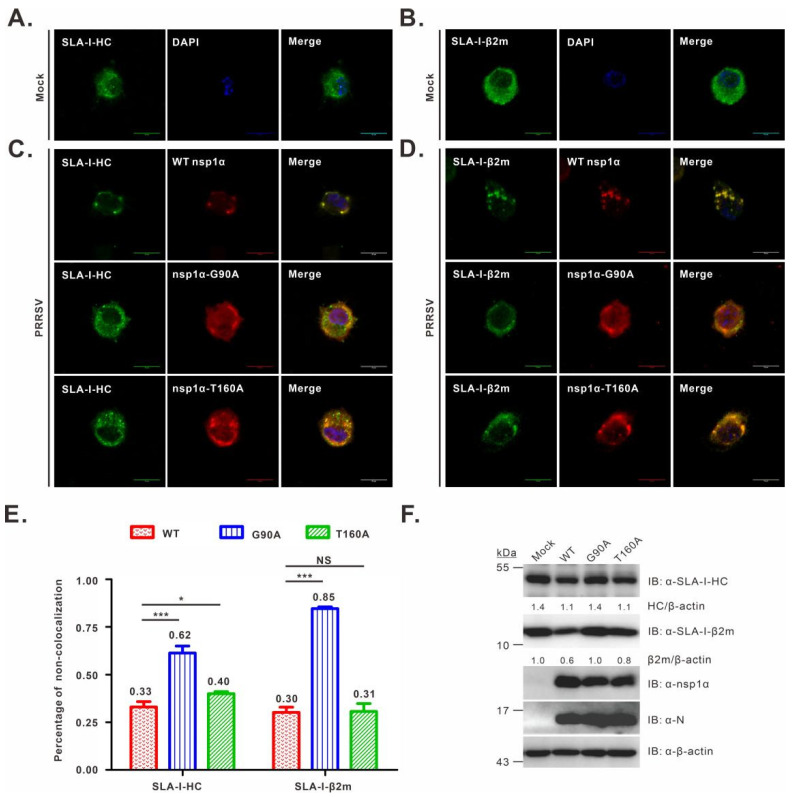
Co-localization analysis of SLA-I with nsp1α or its derivatives in PRRSV-infected PAMs. PAMs grown on coverslips in 12-well plates were mock-infected with RPMI 1640 (**A**,**B**) or infected with the indicated viruses (**C**,**D**) at an MOI of 1.0. At 12 h post-infection, the cells were fixed, permeabilized, and stained with antibodies to SLA-I-HC, SLA-I-β2m, or nsp1α. DAPI was used to stain the cell nuclei. (**E**) The same as above, except that the co-localization relationship was quantified. The graph shows the percentages of cells that exhibited a non-colocalization relationship between nsp1α or its mutants with SLA-I molecules (averages are shown on top of each bar). Asterisks indicate a statistical significance between the WT and the mutants (* *p* < 0.05; *** *p* < 0.001). NS means no significance. The data are shown as the mean ± SD of three independent experiments. (**F**) Western blot analysis of SLA-I abundance in PAMs infected with the indicated viruses (MOI = 1.0) at 12 h post-infection.

It has been shown that nsp1α co-localizes with SLA-I in transfected mammalian cells [27], but it is not known whether this is true in PRRSV-infected PAMs. In addition, it is not clear about the mutational effect on the nsp1α-SLA-I co-localization relationship. To this end, we infected PAMs with WT or the nsp1α mutant viruses, whereas the mock-infection with RPMI 1640 served as a control. We found that in mock-infected cells, SLA-I-HC exhibited a diffusive distribution pattern in the cytoplasm (Figure 4A), whereas in WT-infected cells, it became punctuated and co-localized well with nsp1α (Figure 4C, the upper panel), indicating an active recruitment to the nsp1α site. A similar result was observed for SLA-I-β2m (Figure 4B,D, upper panel). On the other hand, in the cells infected with the nsp1α mutants, nsp1α-G90A co-localized poorly with either SLA-I-HC (Figure 4C, middle panel) or β2m (Figure 4D, middle panel), while nsp1α-T160A showed only partial co-localization with either subunit of SLA-I (Figure 4C, bottom panel; Figure 4D, bottom panel). Further quantitative analyses revealed that the number of cells without co-localization of nsp1α and SLA-I-HC or β2m in the mutant G90A-infected cells showed a significant increase compared to WT (*p* < 0.001), while a moderate ratio was observed for the mutant T160A (Figure 4E). Consistently, Western blot analysis showed a similar expression level of SLA-I in mutant G90A-infected cells compared to that in mock-infected cells, but lower than that in T160A and WT-infected cells (Figure 4F). Thus, it appears that the residue G90 is critical for nsp1α-SLA-I colocalization in infected cells.

### 3.5. PRRSV Strain JXwn06 Carrying the G90A Mutation in nsp1α Failed to Downregulate SLA-I in PAMs

**Figure 5:**
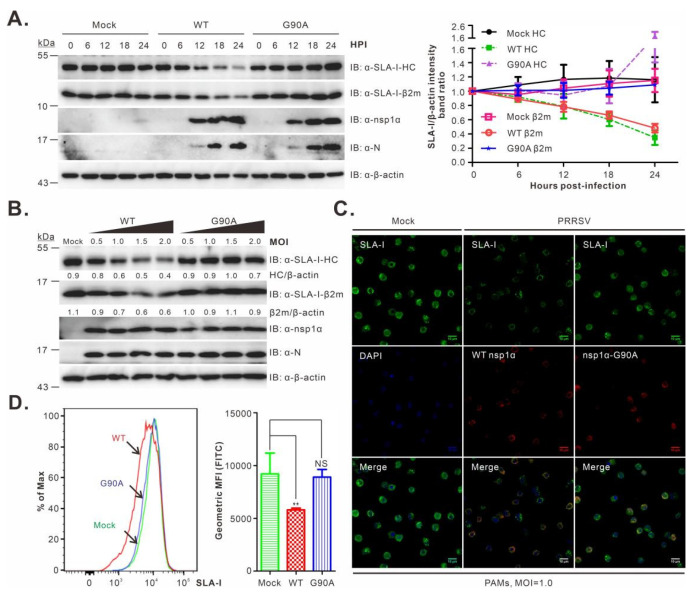
Effect of the nsp1α-G90A mutation on the SLA-I accumulation in virus-infected PAMs. (**A**) PAMs were either mock-infected with RPMI 1640 or infected with rescued viruses WT or the mutant G90A at an MOI of 1.0. At the indicated time points, PAMs were harvested and subject to Western blot analysis using the indicated antibodies. The right graph shows the quantitative analysis of SLA-I abundance measured by the band intensity ratio of SLA-I-HC/β2m over β-actin. (**B**) The same as (**A**), except that different doses were used and that the Western blot analysis was performed at 12 h post-infection. (**C**) The same as (**A**), except that the cells were fixed for IFA analysis at 12 h post-infection with antibodies to SLA-I (JM1E3) and nsp1α. DAPI was used to stain cell nuclei. (**D**) The same as (**A**), except that at 12 h post-infection, PAMs were harvested and subjected to FACS analysis with mouse mAb to SLA-I (JM1E3). The SLA-I levels were presented as the mean fluorescence intensity (MFI) in the right panel. The data shown are the means and standard deviations from three independent experiments (** *p* < 0.01; NS, not significant).

We next investigated the decay of SLA-I in a time-course study. The mutant G90A was chosen for further analysis, as the corresponding mutation exhibited a stronger inhibitory effect on nsp1α activity (Figure 1D,E and Figure 4C,D,F). PAMs were either mock-infected or infected with WT or the mutant G90A at an MOI of 1.0. At different time points post-infection as indicated, the cells were collected and subjected to Western blot analysis. In WT PRRSV-infected cells, a gradual decline in SLA-I-HC was exhibited as the infection progressed, and this became obvious at 12 h post-infection and pronounced at later time points (Figure 5A). A similar trend was observed for SLA-I-β2m (Figure 5A). In contrast, in the cells infected with the mutant G90A, the levels of SLA-I were kept steady (Figure 5A). Moreover, an increase in the infection doses (MOI = 0.5, 1.0, 1.5, or 2.0) did not change the outcome (Figure 5B).

We also examined the cell surface accumulation of SLA-I. In the first assay, PAMs were either mock-infected or infected with WT or mutant viruses and then processed for IFA analysis at 12 h post-infection. Compared to the mock control, WT PRRSV infection led to a clear decrease in SLA-I in the overall fluorescence intensity. In contrast, the infection with the mutant G90A did not have an obvious effect (Figure 5C). We also employed FACS analysis to measure the SLA-I cell surface expression. The results were similar to the IFA analysis; the parental virus infection resulted in a significant shift of the mean fluorescence intensity (*p* < 0.01), whereas the G90A infection exhibited a pattern similar to that of the mock control (*p* > 0.05) (Figure 5D). Together, we concluded that the residue G90 is critical for nsp1α-mediated degradation of SLA-I.

### 3.6. The G90A Mutation of nsp1α Results in Decreased SLA-I Ubiquitination

**Figure 6:**
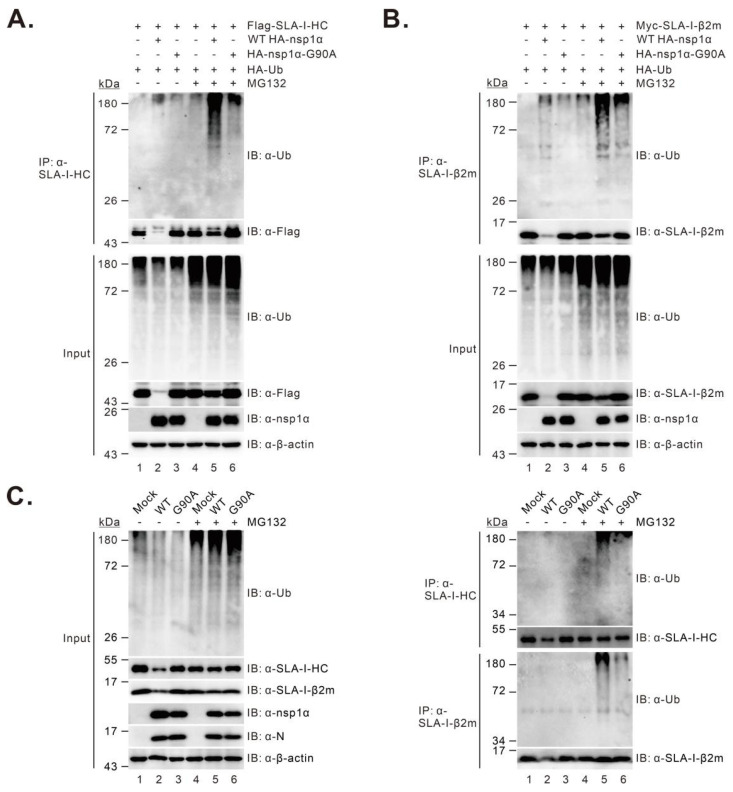
The effect of the G90A mutation on nsp1α-induced SLA-I ubiquitination. (**A**,**B**) HEK 293T cells were co-transfected to express FLAG-SLA-I-HC (**A**) or Myc-β2m (**B**), HA-ubiquitin with or without WT HA-nsp1α or HA-nsp1α-G90A. At 18-24 h post-transfection, the cells were treated with treated with MG132 at a final concentration of 10 μM for 4 h before Co-IP and Western blot analyses with indicated antibodies. β-actin served as a loading control. (**C**) PAMs were mock-infected with RPMI 1640 or infected with 1.0 MOI of the WT or G90A. At 4–6 h post-infection, the cells were treated with 5 μM MG132 for another 8 h before Co-IP and Western blot analyses with the indicated antibodies.

It has been shown that degradation of SLA-I by PRRSV nsp1α depends on the ubiquitin–proteasomal pathway [27]. Thus, we examined the mutational effect of nsp1α on SLA-I ubiquitination in two different assays. In the first assay, HEK 293T cells were transfected to co-express FLAG-SLA-I-HC, Myc-β2m, or HA-ubiquitin with or without WT HA-nsp1α or HA-nsp1α-G90A, and then treated with MG132 at 4 h before collecting for further analysis. The immunoprecipitation (IP) analysis revealed that the level of ubiquitinated SLA-I-HC (Figure 6A, lane 5) or β2m (Figure 6B, lane 5) showed a significant increase in the presence of WT nsp1α compared with that in the control samples (Figure 6A,B, lane 4). In contrast, the expression of nsp1α-G90A did not much affect the ubiquitination of SLA-I-HC (Figure 6A, lane 6, top panel) or β2m (Figure 6B, lane 6, top panel). Similar results were also obtained in the condition of PRRSV infection of PAMs (Figure 6C). Thus, these data suggest that the G90A mutation impairs the ability of nsp1α to mediate SLA-I ubiquitination, providing further evidence for the essential role of Gly90 in the degradation of the SLA-I molecule by nsp1α.

## 4. Discussion

Swine SLA-I plays a critical role in host antiviral immunity by exposing viral antigens to the innate immune cells and initiating the CTL responses [37]. We have previously shown that PRRSV nsp1α is able to induce the proteasomal degradation of SLA-I [27], thus providing a novel perspective on how PRRSV might evade CTL responses. As a follow-up study, this report went on further to unveil critical residues for nsp1α function. Our results here revealed two salient messages: (i) the residues critical for nsp1α function in SLA-I degradation were clustered in a structurally relatively concentrated region, and most of them are critical for PRRSV viability; (ii) the PRRSV strain JXwn06 carrying the nsp1α mutation G90A lost the ability to downregulate SLA-I on the PAMs’ cell surface. The relevant insights and significance are discussed below.

PRRSV nsp1α is a well-known multifunctional replicase protein that participates in multiple aspects of the virus life cycle. During replication, it mediates the co-translational cleavage of itself from the replicase polyprotein pp1a and ppla/b [38,39] and is important for regulating viral subgenomic (sg) mRNA synthesis [40]. It is also a critical modulator of host immunity, including interferon signaling [39,41,42,43,44,45,46,47], inflammation responses [47,48,49], and cellular immunity [27,50]. Notably, one key mechanism for nsp1α-mediated immune modulation is via the degradation of host cellular factors [27,44,46,51,52,53]. A classic target is CREB-binding protein (CBP), a nuclear factor that regulates the activation of many transcriptional factors (e.g., NF-κB and IRF3) as well as the production of some inflammatory cytokines [44,46,52]. The most recently identified substrate is SLA-I, a critical mediator of host cellular immunity [27]. In both cases, degradation depends on the ubiquitin–proteasomal system and requires an intact nsp1α but not the protease activity [27,46]. In this report, we mapped the residues critical for this function of nsp1α. This led to the identification of a total of 11 residues that were found to be localized to both the N-ZF and PCPα domains, consistent with the previous result that an intact molecule is necessary for nsp1α degradation activity [27]. Additionally, most of these residues are essential for PRRSV viability and have not been reported to be associated with any known functions of nsp1α, except for the residues Gly90, Asn91, Arg97, and Asn161 that haven been shown to contribute to the dimerization of nsp1α (Asn91 and Asn161) [30], suppression of IFN signaling (Gly90, Asn91 and Arg97) [45], and downregulation of TNF-α expression (Gly90 and Arg97) [54]. Notably, all identified key residues, except Phe17, are located within the PCPα domain, and eight of them are located within a continuous double alpha helix (aa. 75–100) (Figure 2C), suggesting that this helix might be an important interface for virus–host interactions. Among all the critical residues, the residue Gly90 is quite intriguing, as the mutation of this residue affects all three modulatory functions of nsp1α, including interferon signaling [45], inflammation response [54], and SLA-I degradation (Figure 1C,E and Figure 5A). This convergent effect puts Gly90 in a unique position and suggests that this residue is either important for nsp1α structure or critical for protein–protein interactions or both. Future studies might be directed to further dissect the mechanisms of how nsp1α mediates the degradation of cellular proteins.

Downregulation of SLA-I accumulation on a cell’s surface is a common strategy employed by a variety of viruses to evade a host’s cellular immunity. Many viruses encode proteins to target the MHC-I molecule for proteasomal or lysosomal degradation including HIV (Nef protein) [55], herpesviruses (e.g., ICP47 of herpes simplex virus, US2, US3, US6, and US11 proteins of human cytomegalovirus) [56,57,58], poxvirus (e.g., M153R protein of myxomavirus) [59], and norovirus (e.g., NS3 and VP2 proteins of MNV) [21,60]. Moreover, some viruses, such as murine gamma herpesvirus 68, encode ubiquitin E3 ligases to directly conjugate ubiquitin to the substrate’s MHC-I molecule [61]. These proteins are important targets for vaccine development. For example, the *nef* deletion virus induces higher cellular immune responses than WT virus due to the improved antigen presentation and greater T-cell help [62,63]. The bovine herpesvirus type 1 (BHV-1) mutant lacking the MHC-I downregulation property induces faster onset of cellular immune responses in calves (natural host) [64]. In this study, the G90A mutation disabled the ability of PRRSV to degrade SLA-I in virus-infected PAMs. It will be interesting to investigate whether the same is true in infected pigs and whether the mutant can induce better CTL responses.

## Data Availability

Not applicable.

## Supplementary Materials

The following supporting information can be downloaded at: https://www.mdpi.com/article/10.3390/v14040690/s1, Figure S1: Dose-dependent effect of HA-nsp1α or its derivatives on the level of Flag-SLA-I-HC in transfected cells; Table S1: Effect on SLA-I-HC degradation of the nsp1α mutants with 2–6 alanine substitutions; Table S2: Effect on SLA-I-HC degradation of the nsp1α mutants with 1–3 alanine substitutions; Table S3: Mutational effect of the nsp1α substitutions on viral viability by reverse genetics.
