# Supplementary material for: Mapping the Key Residues within the Porcine Reproductive and Respiratory Syndrome Virus nsp1α Replicase Protein Required for Degradation of Swine Leukocyte Antigen Class I Molecules
Source: Viruses. 2022 Mar 26;14(4):690. doi: 10.3390/v14040690 (PMC9030574; doi:10.3390/v14040690)
Supplement: Supplementary file 1 [file viruses-14-00690-s001.zip › Figure S1.pdf]

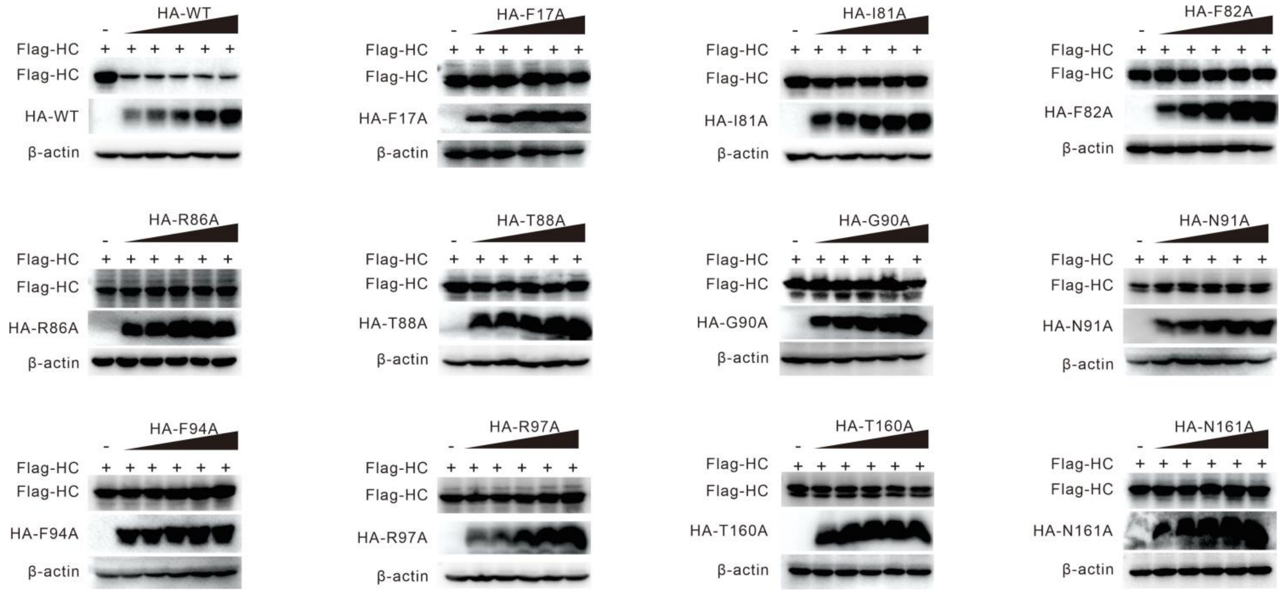

Figure S1. Dose-dependent effect of HA-nsp1α or its derivatives on the level of Flag-SLA-I-HC in transfected cells. HEK 293T cells were transfected to express Flag-SLA-I-HC in combination with an increasing amount (0, 1.0, 1.5, 2.0, 2.5, and 3.0 μg) of a plasmid expressing HA-nsp1α or its mutants. At 36 h post-transfection, the cells were lysed and subject to Western blot analysis with antibodies to either FlAG, nsp1α, or β-actin.
