# Supplementary material for: Mapping the Key Residues within the Porcine Reproductive and Respiratory Syndrome Virus nsp1α Replicase Protein Required for Degradation of Swine Leukocyte Antigen Class I Molecules
Source: Viruses. 2022 Mar 26;14(4):690. doi: 10.3390/v14040690 (PMC9030574; doi:10.3390/v14040690)
Supplement: Supplementary file 1 [file viruses-14-00690-s001.zip › Table S1.pdf]

Table S1. Effect on SLA-I-HC degradation of the nsp1α mutants with 2-6 alanine substitutions by Western blot.

Mutants	Induction of SLA-I-HC degradation	Further mutation
S2-5A	+	No
P12-6A	-	Yes
V18-6A	-	Yes
S30-5A	+	No
L35-5A	+	No
Q40-5A	-	Yes
G45-5A	+	No
F50-5A	-	Yes
E55-5A	+	No
T60-4A	+	No
F65-4A	+	No
P72-3A	+	No
L78-5A	-	Yes
P83-5A	-	Yes
T88-5A	-	Yes
N93-5A	-	Yes
M98-4A	+	No
E104-4A	+	No
G109-5A	-	Yes
T114-5A	+	No
L119-5A	+	No
R124-5A	+	No
Y129-5A	+	No
P134-4A	+	No
V138-4A	-	Yes
N143-2A	+	No
S148-4A	+	No
F152-3A	+	No
T156-5A	-	Yes
N161-5A	-	Yes
Q166-5A	+	No
E171-5A	+	No
F176-5A	+	No
