# Supplementary material for: Mapping the Key Residues within the Porcine Reproductive and Respiratory Syndrome Virus nsp1α Replicase Protein Required for Degradation of Swine Leukocyte Antigen Class I Molecules
Source: Viruses. 2022 Mar 26;14(4):690. doi: 10.3390/v14040690 (PMC9030574; doi:10.3390/v14040690)
Supplement: Supplementary file 1 [file viruses-14-00690-s001.zip › Table S2.pdf]

Table S2. Effect on SLA-I-HC degradation of the nsp1α mutants with 1-3 alanine substitutions by Western blot.

Mutants	Induction of SLA-I-HC degradation	Further mutation
P12-2A	+	No
R15-3A	-	Yes
V18A	+	No
E20-3A	+	No
Q22-2A	+	No
Q40-3A	+	No
E43-2A	+	No
F50-2A	-	Yes
R52-3A	+	No
L78-2A	+	No
I81-2A	-	Yes
P83-2A	+	No
R86-2A	-	Yes
T88-2A	-	Yes
G90-2A	-	Yes
L92-2A	+	No
F94-2A	-	Yes
Q96-2A	-	Yes
G109-2A	+	No
T111-2A	+	No
P113A	+	No
V138-2A	+	No
Y140-2A	+	No
T156-2A	+	No
V158-3A	-	Yes
N161-2A	-	Yes
P163-3A	+	No
