# Supplementary material for: Mapping the Key Residues within the Porcine Reproductive and Respiratory Syndrome Virus nsp1α Replicase Protein Required for Degradation of Swine Leukocyte Antigen Class I Molecules
Source: Viruses. 2022 Mar 26;14(4):690. doi: 10.3390/v14040690 (PMC9030574; doi:10.3390/v14040690)
Supplement: Supplementary file 1 [file viruses-14-00690-s001.zip › Table S3.pdf]

Table S3. Mutational effect of the nsp1α substitutions on viral viability by reverse genetics.

Mutants	CPE	Anti-N immunofluorescence	Virus viability
F17A	-	-	No
I81A	-	-	No
F82A	-	-	No
R86A	-	-	No
T88A	-	-	No
G90A	+	+	Yes
N91A	-	-	No
F94A	-	-	No
R97A	-	-	No
T160A	+	+	Yes
N16A	-	-	No
